# Gene expression profiling and histopathological characterization of triple-negative/basal-like breast carcinomas

**DOI:** 10.1186/bcr1771

**Published:** 2007-10-02

**Authors:** Bas Kreike, Marieke van Kouwenhove, Hugo Horlings, Britta Weigelt, Hans Peterse, Harry Bartelink, Marc J van de Vijver

**Affiliations:** 1 Division of Radiation Oncology, The Netherlands Cancer Institute, Plesmanlaan 121, 1066 CX Amsterdam, The Netherlands; 2 Division of Experimental Therapy, The Netherlands Cancer Institute, Plesmanlaan 121, 1066 CX Amsterdam, The Netherlands; 3 Division of Diagnostic Oncology, The Netherlands Cancer Institute, Plesmanlaan 121, 1066 CX Amsterdam, The Netherlands

## Abstract

**Introduction:**

Breast cancer is a heterogeneous group of tumors, and can be subdivided on the basis of histopathological features, genetic alterations and gene-expression profiles. One well-defined subtype of breast cancer is characterized by a lack of *HER2 *gene amplification and estrogen and progesterone receptor expression ('triple-negative tumors'). We examined the histopathological and gene-expression profile of triple-negative tumors to define subgroups with specific characteristics, including risk of developing distant metastases.

**Methods:**

97 triple-negative tumors were selected from the fresh-frozen tissue bank of the Netherlands Cancer Institute, and gene-expression profiles were generated using 35K oligonucleotide microarrays. In addition, histopathological and immunohistochemical characterization was performed, and the findings were associated to clinical features.

**Results:**

All triple-negative tumors were classified as basal-like tumors on the basis of their overall gene-expression profile. Hierarchical cluster analysis revealed five distinct subgroups of triple-negative breast cancers. Multivariable analysis showed that a large amount of lymphocytic infiltrate (HR = 0.30, 95% CI 0.09–0.96) and absence of central fibrosis in the tumors (HR = 0.14, 95% CI 0.03–0.62) were associated with distant metastasis-free survival.

**Conclusion:**

Triple-negative tumors are synonymous with basal-like tumors, and can be identified by immunohistochemistry. Based on gene-expression profiling, basal-like tumors are still heterogeneous and can be subdivided into at least five distinct subgroups. The development of distant metastasis in basal-like tumors is associated with the presence of central fibrosis and a small amount of lymphocytic infiltrate.

## Introduction

The World Health Organization has defined a wide range of histopathological subtypes of invasive breast cancer and classified these carcinomas into 19 categories [1], most of which are quite rare [2]. This classification into subtypes of tumors is based on histopathological characteristics, but does not reflect disease outcome. Perou *et al*. and Sorlie *et al*. were the first to show that breast carcinomas can also be subdivided based on gene-expression analysis [3-6]. They have used hierarchical cluster analysis based on the expression pattern of a set of genes, termed the 'intrinsic gene subset'. Using this approach, breast carcinomas can be subdivided into several subgroups that differ in their overall gene-expression profile. The largest difference in overall gene-expression profile is observed between tumors that are estrogen receptor (ER) positive and those that are ER negative [4]. These ER-negative tumors are further sub-divided into tumors with gene characteristics of *HER2*-positive tumors, normal breast tissue and basal epithelial/myoepithelial cells. These subgroups were called 'the molecular subtypes' and were originally based on an intrinsic gene set derived from 65 tissue samples from 42 individuals [4].

Many of the genes characteristic for breast myoepithelial/basal epithelial cells were highly expressed in a group of six tumors. To confirm the basal-like characteristic of this group, immunohistochemistry was performed with antibodies against breast basal cell keratins 5/6 and 17, for which all six basal-like tumors stained positive. These six tumors were further characterized by lack of expression of *ER *and absence of *HER2 *gene amplification, and are associated with poor survival [3-6]. In subsequent investigations, Perou *et al*. studied larger series of tumors (*n *= 416 cases) and refined the composition of the intrinsic gene set [3,5,6].

Additional efforts have been made to characterize these basal-like tumors with standard histopathology and immunohistochemical analyses [7,8]. Nielsen *et al*. identified a panel of antibodies (ER, epidermal growth factor receptor (EGFR), HER2 and KRT 5/6) that could accurately discriminate basal-like tumors from the other molecular subtypes. They used a panel of 21 basal-like tumors defined by gene-expression profiling, and correlated the immunohistochemical features to those of a series of 663 breast tumors. They found that 15% were of the basal subtype, staining negative for ER, progesterone receptor (PR) and HER2 in all cases and positive for KRT 5/6 and/or EGFR in all cases [8]. Kim *et al*. studied 776 breast tumors by immunohistochemistry, and subdivided this group into five groups based on the pattern of marker expression. Basal-like tumors were defined by staining negative for ER, PR and HER2, and positive for KRT5 and/or KRT14 and/or EGFR and/or KIT [7]. It is believed that basal-like tumors constitute a homogenous sub-group of breast carcinomas [3-8].

ER-negative breast carcinomas in general are associated with relatively poor prognosis [9-11]; based on published series, these patients have a 10 year relapse-free survival of 55–70%. As these tumors are ER-negative, these patients are not treated with adjuvant endocrine treatment but often undergo adjuvant chemotherapy treatment. If the 55–70% of patients with ER-negative breast cancer that will not develop distant metastases can be accurately identified, these patients could be spared adjuvant chemotherapy treatment. We have previously identified a 70-gene prognosis profile [12,13]. As nearly all ER-negative tumors show a poor prognosis profile, this 70-gene profile is not suitable to identify good and poor prognosis subgroups within the category of ER-negative breast cancer patients. Wang *et al*. have performed gene-expression profiling of a series 35 ER-negative breast carcinomas [14]. They identified a 16-gene prognosis profile with the capacity to distinguish ER-negative breast carcinomas with good or poor survival outcome; however, these tumors were not all triple negative.

We examined the histopathological features and overall gene-expression profile of a large group of triple-negative tumors. We explore how homogeneous the overall gene-expression profile is *within *the group of basal-like tumors. In addition, we test whether we can identify subsets of tumors defined by distinct differences in gene-expression patterns or histopathological features, including subsets associated with a low risk of developing distant metastases.

## Materials and methods

### Selection of triple-negative tumors

We selected breast carcinomas from patients treated between January 1985 and February 2005 at the Netherlands Cancer Institute. Based on pathology reports we identified tumors that were reported to lack immunohistochemical expression of ER, progesterone receptor (PR) and HER2 (triple-negative status). We linked a database containing these tumors with the database of the fresh-frozen tissue bank of the Netherlands Cancer Institute, and selected 97 tumors with a triple-negative status of which frozen material was present.

For 71 out of 97 patients we had clinical follow-up data available (median 5.1 years, range 0.3–17.8). All 71 patients had no prior malignancies (excluding non-melanoma skin cancer and dysplasia of the uterine cervix), and did not receive any systemic therapy before surgery. Therapy for the 71 patients consisted of breast conserving surgery or modified radical mastectomy with axillary lymph node dissection or sentinel lymph node procedure. In 52 cases local therapy was followed by systemic therapy with either chemotherapy (*n *= 37; 17/37 Anthracyclin-based and 20/37 Cyclophosphamide, Methotrexate and Fluorouracil (CMF)), endocrine treatment (*n *= 11 Tamoxifen) or a combination of both modalities (*n *= 4; 2/4 Anthracyclin-based and Tamoxifen, and 2/4 CMF and Tamoxifen).

All patients were informed that tumor tissue was stored for future research purposes unless the individual patient made an objection to this. The medical ethical committee of the Netherlands Cancer Institute approved this study.

### Control tumors (not triple negative)

In addition to 97 triple-negative tumors, we also used gene-expression data from 102 invasive breast carcinomas that were part of an unrelated research project in our institute and that included ER- and/or PR- and/or HER2-positive tumors. These two gene-expression datasets differ only in sample identity, but are similar with regards to patient characteristics and experimental work-up. We used these two datasets in order to perform unsupervised hierarchical cluster analysis of triple-negative tumors in combination with all other tumor types, enabling us to observe to what extent triple-negative tumors cluster together based on their overall gene-expression profile.

### Characterization of tumors by histology and immunohistochemistry

For all cases an original pathological assessment was done in the pathology report, and immunohistochemistry was performed for all cases. In this study, immunohistochemistry was repeated and all cases were evaluated by one pathologist (MV) in a standardized fashion; usually between 2–6 slides per tumor were available for re-evaluation. The features scored included: tumor diameter, histological type, grade, presence and amount of vascular invasion, amount and type of a ductal carcinoma *in situ *component, amount of lymphocytic infiltrate and presence of central fibrosis. The amount of lymphocytic infiltrate was scored as follows: none = no lymphocytes present; minimal = scattered lymphocytes, <10 lymphocytes per high power field (40x); moderate = lymphocytes easily identified, but no large aggregates; extensive = large aggregates of lymphocytes in >50% of the tumor. Central fibrosis was deemed to be present when the center of the tumor showed collagen, with a variable amount of fibroblasts, without tumor cells. Our series includes four adenoid cystic carcinomas. As these tumors are a separate entity of basal-like tumors, some histopathological characteristics were not scored. Immunohistochemical staining was performed on paraffin sections from 95 specimens (for two tumors, no paraffin-embedded tumor tissue could be retrieved). Sections were stained with antibodies against ER (1D5+6F11; dilution 1:50; Neomarkers, Lab Vision Corporation, Fremont, California, USA); PR (PR-1, dilution 1:400; Klinipath, Duiven, Netherlands), HER2 (3B5; dilution 1:3,000, Neomarkers), p53 (D07; dilution 1:6,000; Dako, Glostrup, Denmark), KRT5/6 (D5/16 B4; dilution 1:100; Dako), KIT (CD117; dilution 1:100; Dako) and EGFR (111.6; dilution 1:100; Neomarkers). Details on the immunohistochemical methods used were previously described by Hannemann *et al*. [15]. Immunohistochemical results were scored semiquantitatively. Tumors were considered positive for hormone receptors if at least 1% of the tumor cells showed nuclear staining. Staining for HER2 was scored according to the clinical guidelines for the assessment of HER2 status: 0, no staining; 1+, more than 10% of cells showed weak and incomplete membrane staining; 2+, moderately strong membrane staining in >10% of the tumor cells; 3+, strong membrane staining in >10% of the tumor cells. A score of 2+ was followed by additional CISH-analysis to assess *HER2 *gene amplification. Tumors were considered positive for p53 if at least 50% of the tumor cells showed nuclear staining and tumors were considered positive for KIT, KRT5/6 or EGFR if at least 1% of the tumor cells showed staining. The cutoff of 1% was selected on the basis of previous studies performed by others [7,8].

### Freezing of tumor samples, RNA isolation and microarray analysis

Tissue samples were snap frozen in liquid nitrogen within one hour after surgery. Sections were cut from these frozen tissue blocks for RNA isolation. The first and the last section were used to assess the percentage of tumor cells by HE staining; only tumors containing an average of >50% tumor cells were used in this analysis. Total RNA was isolated with Trizol (Invitrogen, Breda, Netherlands) and dissolved in RNase-free water. The RNA was treated with DNase; 2 μg of RNA was amplified and 1 μg of aRNA was used for hybridization on the microarray; detailed information on protocols can be found at the central microarray facility website of the Netherlands Cancer Institute [16].

We used the Human Genome Oligo Set Version 3.0 arrays containing 34,580 probes representing 24,650 genes. These arrays were obtained from the central microarray facility at the Netherlands Cancer Institute; detailed information on these arrays can be found at the central microarray facility website of the Netherlands Cancer Institute [16]. RNA from tumor samples was co-hybridized with reference RNA isolated from a reference pool consisting of over 100 breast cancer samples. For all tumors, hybridization was also repeated after reverse color labelling. Fluorescent intensities were normalized and corrected for biases as previously described by Hannemann *et al*. [17] and weighted averages, and confidence levels were computed according to the Rosetta Error Model [18]. Gene-expression data are publicly available at ArrayExpress, accession number E-NCMF-2 [19].

### Data analysis

A subset of the total of 34,580 probes was selected, based on the following criteria: expression data should be available for at least 99% of all experiments and the expression level should be significantly different from the reference expression in at least 19 experiments with a *P *value of < 0.01. These criteria reduced the total number of genes from 34,580 to 7,770. As the differences between gene expression in the study group and the reference pool are larger than the variation within the study group, the intensity ratios were converted with respect to the mean expression of each gene within the study group.

Unsupervised and supervised methods of analysis were performed. We have used the intrinsic gene subset described previously by Perou *et al*. and Sorlie *et al*. [3-6] to define basal-like, luminal A-like, luminal B-like, ERBB2-like and normal breast-like tumor classes on the basis of hierarchical clustering and correlation to the class centroids; using the intrinsic gene list as recently updated by Hu *et al*. [3]. We identified almost all intrinsic genes on our microarray platform (293 out of 306 unique genes).

We performed average-linkage hierarchical clustering of an uncentered Pearson correlation similarity matrix of the 97 primary tumors with 7,770 filtered genes with the program Cluster, and results were visualized with TreeView [20]. Over-representation of genes representing specific Gene Ontology (GO) categories in specific gene clusters were identified with the software EASE [21].

Supervised classification was performed on the 71 samples with follow-up data using SAM-software [22] developed by Tusher *et al*. We used the settings in the software for censored survival data. This approach was fitted to select genes that are differently expressed between patients with and without distant metastasis as first event during the follow-up period. A threshold was chosen that reflects the lowest median false-discovery rate as estimated after repeatedly permuting (1,000 times) the labels and counting the number of genes that were called significant at each threshold. In addition to the analyses with the SAM software we also used PAM software [23] developed by Tibshirani *et al*. for class prediction analyses using the shrunken-centroid technique.

The relationship of the pathological information and the gene-expression profiles was studied by cross tabulation with chi-square tests, Kaplan-Meier with log-rank test and proportional hazard Cox regression analyses, using SPSS software version 12 (Chicago, Illinois, USA). All variables in the equation were used as ordinal variables. Univariate tests were considered significant at a level of *P *< 0.05.

## Results

### Clinical and histopathological features

We have selected 97 breast carcinomas that were shown to be ER negative, PR negative and HER2 negative by immunohistochemical staining. The clinical and pathological features of these 97 tumors are summarized in Table 1. As can be seen, the 97 triple-negative tumors were classified as invasive ductal carcinomas in 83% of the cases; 78% had sharply demarcated borders; 87% were histological grade 3, 46% contained a moderate to extensive lymphocytic infiltrate and 51% of the tumors contained a central fibrotic zone. The immunohistochemical analysis showed that 76% of tumors stained positive for KRT 5/6, 27% were positive for EGFR and 38% stained positive for KIT; 50% of the tumors were positive for p53 staining (*TP53 *mutation analysis was not carried out).

**Table 1:** Pathological and clinical characteristics

Variable	All triple negatives; *n *= 97 (%)	Triple negatives with sufficient FU; *n *= 71 (%)
***Age (years)***		
<41	24 (25)	18 (25)
41–50	33 (34)	30 (42)
>50	40 (41)	23 (32)
***Tumor size (cm)***		
≤2.0	34 (35)	23 (32)
>2.0	63 (65)	48 (68)
***Number of tumor positive lymph nodes***		
0	54 (56)	35 (49)
1–3	29 (30)	26 (37)
>3	14 (14)	10 (14)
***Tumor subtype***		
Adenoid cystic	4 (4)	NA
Apocrine	7 (7)	7 (10)
Ducto-lobular	1 (1)	NA
IDC (nos)	81 (83)	63 (89)
ILC	1 (1)	1 (1)
Metaplastic	3 (3)	NA
***Tumor shape***		
Multi-nodular	4 (4)	4 (6)
Stellate	17 (18)	12 (17)
Sharply demarcated/lobulated	76 (78)	55 (76)
***Histological grade***		
1	2 (2)	2 (3)
2	8 (8)	4 (6)
3	83 (86)	65 (92)
Adenoid cystic carcinoma not graded	4 (4)	NA
***DCIS component around tumor***		
None	79 (81)	56 (79)
Minimal	10 (10)	8 (11)
Moderate	8 (8)	7 (10)
Extensive	NA	NA
***DCIS differentiation grade***		
Well	1 (6)	1 (7)
Moderate	NA	NA
Poor	17 (94)	14 (93)
***LCIS component***		
None	93 (96)	67 (94)
Present	4 (4)	4 (6)
***Vascular invasion***		
None	84 (87)	59 (83)
Present	13 (13)	12 (17)
***Lymphocytic infiltrate***		
None	8 (8)	4 (6)
Minimal	40 (41)	34 (48)
Moderate	33 (34)	25 (35)
Extensive	12 (12)	8 (11)
Adenoid cystic carcinoma not graded	4 (4)	NA
***Central fibrotic zone***		
None	44 (45)	31 (44)
Present	49 (51)	40 (56)
Adenoid cystic carcinoma not graded	4 (4)	NA
***KRT5/6***		
Negative (0% staining)	21 (22)	16 (23)
Positive (> 0% staining)	74 (76)	53 (75)
Unknown	2 (2)	2 (3)
***p53***		
Negative (< 50% staining)	47 (49)	33 (47)
Positive (> 49% staining)	48 (50)	36 (51)
Unknown	2 (2)	2 (3)
***EGFR***		
Negative (0% staining)	69 (71)	58 (82)
Positive (> 0% staining)	26 (27)	11 (16)
Unknown	2 (2)	2 (3)
***KIT***		
Negative (0% staining)	58 (60)	39 (55)
Positive (> 0% staining)	37 (38)	30 (42)
Unknown	2 (2)	2 (3)
***Treatment of primary tumor***		
Breast-conservation	61 (63)	45 (63)
Mastectomy	36 (37)	26 (37)
***Adjuvant radiotherapy***		
Yes	85 (88)	65 (92)
No	12 (12)	6 (8)
***Adjuvant systemic therapy***		
Chemotherapy	47 (48)	37 (52)
Endocrine therapy	13 (13)	11 (15)
Chemotherapy and endocrine therapy	4 (4)	4 (6)
None	33 (34)	19 (27)
***Metastasis as first event***		
Yes	NA	17 (24)
No	NA	54 (76)

DCIS, ductal carcinoma *in situ*; EGFR, epidermal growth factor receptor; FU, follow-up; IDC (nos), infiltrating ductal carcinoma (not otherwise specified); ILC, infiltrating lobular carcinoma; LCIS, lobular carcinoma *in situ*; NA, not applicable.

### Gene-expression profiles of 97 triple-negative tumors

For each of the 97 tumors the expression of 24,650 genes was assessed by microarray analysis. First, we wished to assess the association of the gene-expression profile of our tumors to that of basal-like tumors as defined by Perou *et al*. [3]. For this purpose we calculated the correlation coefficient between the gene-expression profiles of the 97 triple-negative tumors and the centroids of the five molecular subtypes as described previously by Hu *et al*. [3]. Tumors were assigned to one of the molecular subtypes based on the highest correlation coefficient between their expression profile and the five individual centroids of the molecular subtypes (that is, basal-like, ERBB2-like, normal breast-like, luminal A and luminal B). Tumors were scored as unclassifiable when the correlation coefficient to any of the molecular subtypes was lower than 0.1. Using this approach, 88 triple-negative tumors (91%) were classified as basal-like; of the remaining 9 tumors, 5 were unclassifiable and 4 were assigned to the normal breast-like subtype. These 4 normal breast-like tumors included one infiltrating lobular carcinoma, one ducto-lobular carcinoma and two adenoid cystic carcinomas. The gene-expression pattern of these two adenoid cystic carcinomas had very high correlation coefficients to the basal-like centroid (both 0.30), but the correlation coefficient to the normal breast-like centroid was slightly higher (0.33 and 0.41, respectively).

**Figure 1:**
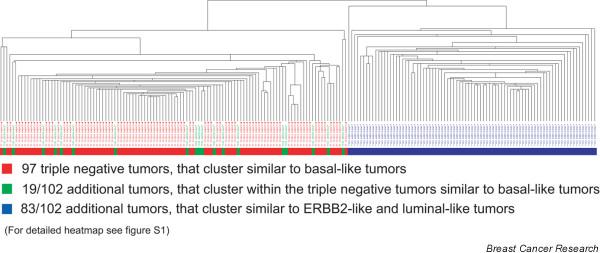
Tumor dendogram of 199 samples. The dendogram depicts the hierarchical cluster analysis with 97 triple-negative tumors (red) combined with 102 invasive breast carcinomas, which were not selected based on their triple-negative status (green and blue), using the 293 intrinsic-gene list.

In order to investigate how the gene-expression profiles of triple-negative tumors is related to basal-like tumors when analysed together with ER-, PR- and/or HER2-positive tumors, we performed hierarchical cluster analysis with the 97 triple-negative tumors combined with 102 invasive breast carcinomas, that were not selected based on their triple-negative status, using the 293 intrinsic-gene list [3] (Figure 1 and Additional file 1). This shows that all triple-negative tumors cluster together as basal-like tumors, separate from the ERBB2-like and luminal-like tumors. Among these 102 additional tumors were 19 tumors that also clustered with the basal-like tumors. All nine triple-negative tumors that were not classified as basal-like, based on the correlation coefficient to the basal-like centroid, clustered together with the basal-like tumors. These data indicate that it is reasonable to define basal-like tumors as those tumors that are negative for ER, PR and HER2 by immunohistochemistry, and that gene-expression profiling or additional immunohistochemical markers are not a requirement to identify these basal-like tumors.

**Figure 2:**
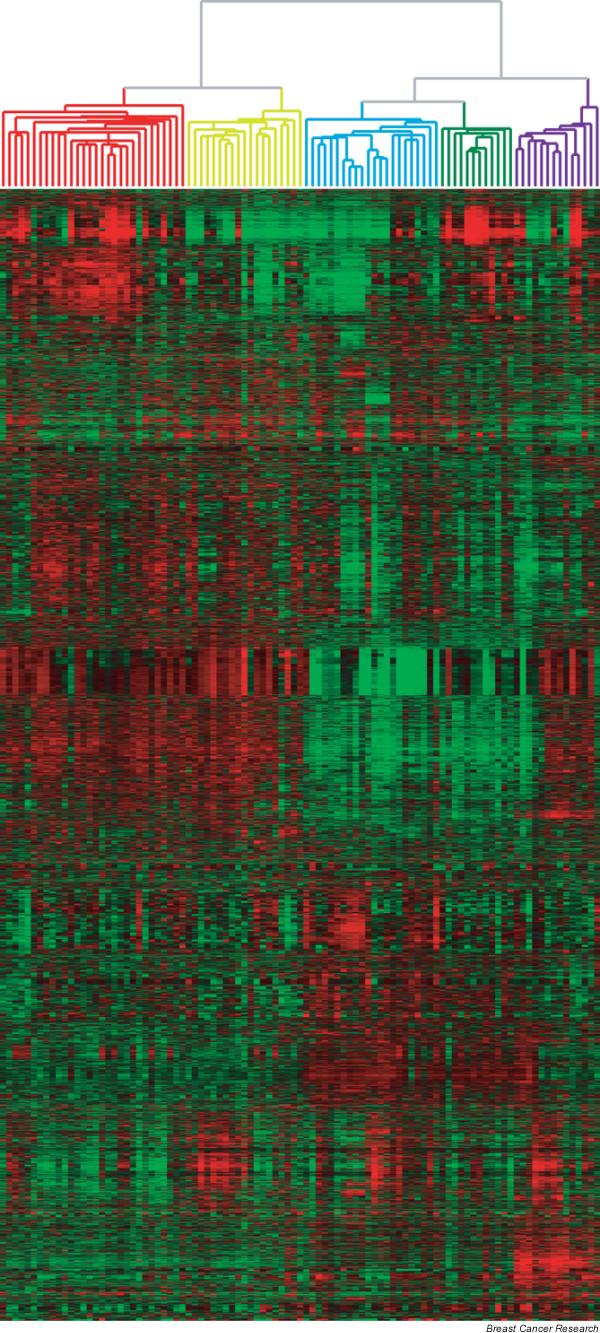
Overall gene expression profile of triple-negative tumors. Hierarchical cluster analysis of 97 triple-negative tumors and 7,770 genes.

To further explore differences in gene expression among the 97 triple-negative tumors, we performed unsupervised hierarchical clustering with 7,770 significantly regulated genes (Figure 2). In Additional file 2 we have highlighted several gene clusters that could be identified. Gene clusters that are represented by basal keratins (cytokeratins 5, 6, 12, 16 and 17; Additional file 2 part d); genes associated with proliferation, including *BUB1, BIRC5, H2AFZ, CCNA2 *and *CDC2 *(Additional file 2 part e) [3]; genes previously described by Farmer *et al*. representing an apocrine-luminal gene cluster [24] that includes *AR, FASN *and *MSX2 *(Additional file 2 part h) and an apocrine-basal gene cluster including *EGFR, CLDN1 *and *VLDLR *[24] (Additional file 2 part g). The interferon-regulated genes *STAT1, CASP1, IFIH1 *and *CXCL10 *cluster together (Additional file 2 part c) [3], and the expression of the genes in this cluster is highly correlated with the expression of genes in the immunoglobulin gene cluster that includes *IGHG1, IGHG3, IGLL1 *and *IGHV1-69 *(Additional file 2 part b).

In addition to these known gene clusters we have also tried to denominate the remaining unknown clusters of genes using EASE-software. We compared each cluster of genes to the entire gene set of 7,770 genes, and looked at whether the gene cluster was enriched with genes that belong to a particular GO category. With this procedure we could identify a gene cluster that holds an over-representation of genes belonging to 'ion transporter activity', including *ASNA1, NDUFS8, NDUFV1 *and *NDUFB7 *(Additional file 2 part f). The genes involved in the remaining clusters have a heterogeneous GO annotation, and it is therefore not possible to assign a uniform biological mechanism to the expression pattern of these individual clusters.

As a next step, we investigated whether tumors clustering together based on their gene-expression profile were characterized by specific pathological characteristics listed in Table 1. The partitioning of the 97 tumors in two main groups (left versus right branch) is associated with a non-random distribution of immunohistochemical staining for EGFR and KIT; in the left branch 87% of the tumors are EGFR negative versus 58% of the tumors in the right branch (*P *= 0.002), and 43% versus 79% of the tumors in these clusters were negative for KIT (*P *= 0.0003).

**Figure 3:**
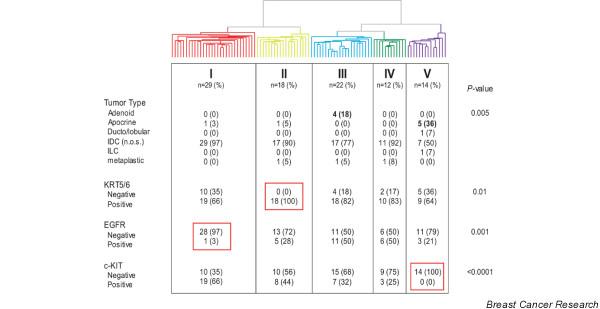
Association of pathological and immunohistochemical characteristics with the overall gene-expression profile of triple-negative tumors. Distribution of tumor type and immunohistochemical staining for KRT5/6, epidermal growth factor receptor (EGFR) and KIT in the dendogram of the 97 triple-negative tumors after hierarchical clustering of these samples based on the expression of 7,770 genes.

The subdivision of tumors into 5 sub-branches (Figure 3) shows a non-random distribution of tumor subtypes and immunohistochemical staining of KRT5/6, EGFR and KIT. All 4 adenoid cystic carcinomas cluster together in branch III and 5/7 of the apocrine tumors cluster together in branch V. All 18 tumors in branch II are positive for KRT5/6 staining, 97% of the tumors in branch I are EGFR negative, and all 14 tumors in branch V are KIT negative. These KIT-negative tumors are further characterized by homogenous overexpression of *AR*.

One of the features from Table 1 that is not correlated to the subdivision in 5 branches is the amount of lymphocytic infiltrate (*P *= 0.09). However, higher expression levels of interferon-regulated and immunoglobulin genes are correlated to larger amount of lymphocytic infiltrate (*P *= 0.01, data not shown).

### Identification of prognostic subgroups

**Figure 4:**
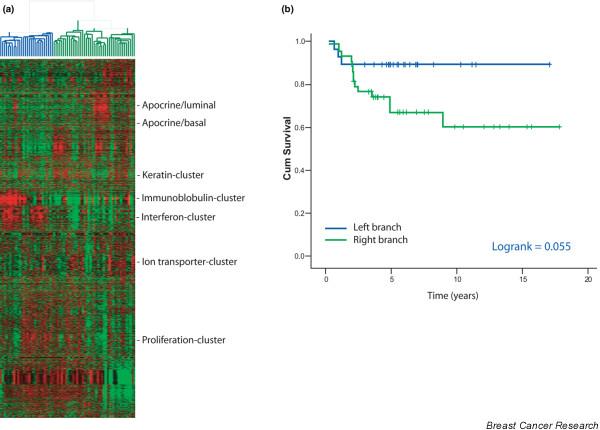
Overall gene-expression profile of triple-negative tumors associated with disease outcome. (a) Hierarchical cluster analysis of the overall gene-expression profile of 71 triple-negative tumors with identifiable gene clusters indicated on the right hand side. (b) Metastasis-free survival of 71 patients with triple-negative breast carcinomas, comparing the left branch with the right branch of the overall gene-expression hierarchical cluster analysis.

For 71 patients long-term clinical follow-up information (median 5.1 years, range 0.3–17.8) was available (detailed patient characteristics are provided in Table 1). The 5-year metastasis-free survival for these 71 patients is 74% (Additional file 3). Most patients were treated by breast conserving surgery, followed by radiotherapy and systemic therapy. Although the tumors were all ER-negative, 21% of the patients were treated with Tamoxifen. In the past Tamoxifen use was not restricted to ER-positive patients only; these guidelines have changed since the patients in this study were treated. Unsupervised hierarchical clustering of these 71 patients with the filtered gene set shows a similar overall gene-expression profile as described for the whole series of 97 tumors. The unsupervised hierarchical clustering of these tumors results in two main branches; the distribution of metastases as first event between these two main branches shows a borderline significant difference; 3/28 (11%) patients in the left branch developed distant metastases versus 14/43 in the right branch (33%), *P *value = 0.047. Kaplan-Meier analysis for metastasis-free survival shows a trend that tumors from the left branch are associated with better survival than the right branch, *P *value = 0.055 (Figure 4). Tumors from the left branch are characterized by their higher expression levels of interferon-regulated and immunoglobulin genes. The mean expression level for the interferon-regulated gene cluster is above the mean of the entire study population in 24/28 (86%) tumors in the left branch versus 12/43 (28%) in the right branch (*P *< 0.00001), and these numbers for the immunoglobulin gene cluster are 21/28 (75%) versus 15/43 (35%), *P *= 0.0014.

Subsequently, we performed supervised classification comparing tumors from patients that developed distant metastases as first event (*n *= 17) with those from patients that remained free of distant metastases as first event (*n *= 54). We used SAM software to look for genes that are differently expressed between the two groups; this resulted in a lowest median false discovery rate of 97% after 1,000 permutations (Additional file 4). We applied PAM software for class prediction and used the gene set with the lowest training error for cross validation; this resulted in an overall misclassification error of 45%. These high false discovery rates indicate that the supervised approaches applied cannot reveal significant differences in gene expression associated with distant metastases.

**Figure 5:**
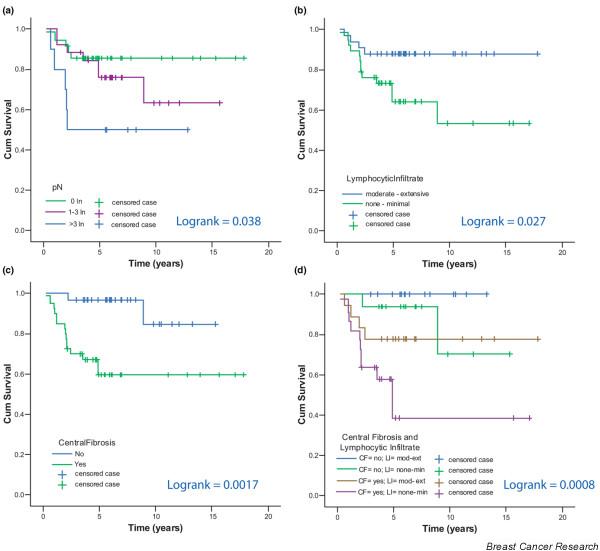
Kaplan-Meier curves. Metastasis-free survival of 71 patients with triple-negative breast carcinomas comparing: (a) number of tumor-positive lymph nodes, (b) amount of lymphocytic infiltrate, (c) presence of a central fibrotic zone and (d) combination of the amount of lymphocytic infiltrate and presence of a central fibrotic zone.

We also studied the relationship of histopathological variables (Table 1) with metastasis-free survival. The number of tumor-positive lymph nodes, the amount of lymphocytic infiltrate and the presence of a central fibrotic zone were associated with disease outcome. The 5-year metastasis-free survival for patients without tumor-involved lymph nodes was 86%; for patients with 1–3 involved nodes this was 76% and for patients with more than 3 involved nodes 50% (log rank = 0.038, Figure 5a). The 5-year metastasis-free survival for patients with a moderate or extensive amount of lymphocytic infiltrate in their tumors compared with those with none or minimal lymphocytic infiltrate was 88% versus 64% (*P *value = 0.027, Figure 5b). Moreover, none of the patients (*n *= 8) with an extensive component of lymphocytic infiltrate developed distant metastases. The 5-year metastasis-free survival for patients with no central fibrosis in their tumors compared with those with central fibrosis is 97% versus 60% (log rank = 0.0017, Figure 5c). Four subgroups can be distinguished on the basis of the amount of lymphocytic infiltrate and central fibrosis. All patients with moderate-extensive lymphocytic infiltrate in combination with no central fibrotic zone remained free of metastases; patients with tumors containing none-minimal lymphocytic infiltrate in combination with a central fibrotic zone had a 5-year metastasis-free survival of 39%, and those with moderate-extensive lymphocytic infiltrate in combination with a central fibrotic zone had a 78% 5-year metastasis-free survival, and those with none-minimal lymphocytic infiltrate in combination with no central fibrotic zone had a 94% 5-year metastasis-free survival (*P *= 0.0008, Figure 5d).

We performed a multivariable Cox proportional hazard analysis to identify independent risk factors for metastasis-free survival. Owing to our relatively small dataset we did not test all variables from Table 1 in the Cox model, but only used those variables that had a *P *value < 0.15 in the univariable analysis, which included: central fibrosis, lymphocytic infiltrate, number of tumor-positive lymph nodes, partitioning of samples by hierarchical clustering into two main groups and tumor diameter. This analysis revealed that only lymphocytic infiltrate (*P *= 0.043) and central fibrosis (*P *= 0.010) are independent risk factors for metastasis-free survival in patients with triple-negative tumors. The hazard rate for distant metastasis in tumors without presence of central fibrosis versus those with central fibrosis was 0.14 (95% CI = 0.030–0.62), and 0.30 for the presence of moderate or extensive amounts of lymphocytic infiltrate versus none or minimal lymphocytic infiltrate (95% CI = 0.090–0.96).

### Comparison of the Rotterdam ER-negative 16-gene signature

Wang *et al*. have identified a 16-gene signature associated with distant metastasis in ER-negative breast carcinomas [14]. We were able to map all 16 genes to our microarray platform and performed hierarchical cluster analysis based on the expression of these 16 genes for the series of 71 tumors (Additional file 5 part a). We observed two main groups of tumors based on the overall expression pattern of these 16 genes. One group consists of 30 tumors (left branch) and the other group of 41 tumors (right branch), with 9 (30%) and 8 (11%) patients who developed distant metastases (*P *= 0.4), respectively. We performed a Kaplan-Meier analysis comparing the left branch with the right branch and found no significant difference in metastasis-free survival (log rank = 0.40, Additional file 5 part b).

## Discussion

We have performed gene-expression profiling in a series of 97 invasive breast carcinomas that were all negative for the expression of ER, PR and HER2 ('triple negative tumors'). This category of breast carcinomas has been shown to stand out from the other breast cancer subtypes by a unique gene-expression profile (basal-like subtype) revealed by gene-expression profiling studies [3-6,24-26].

Immunohistochemical analyses of these tumors have shown that the tumor cells frequently express KRT5/6, EGFR and KIT [7,8].

There is some confusion in the literature as to what defines a basal-like tumor. The term was introduced by Perou *et al*. [4] as describing a subgroup of tumors that was defined by their great similarity in overall gene-expression pattern of the 'intrinsic gene subset' when unsupervised hierarchical clustering was applied. As outlined above, several studies have indicated that these basal-like tumors have low mRNA expression of the *ER, PR *and *HER2 *genes, and are usually also negative for expression of ER, PR and HER2 measured using immunohistochemistry. In our study, we have selected tumors based on the absence of immunohistochemical staining for ER, PR and HER2. When these tumors are analysed by unsupervised hierarchical cluster analysis using the intrinsic gene subset together with tumors that are positive for ER, PR and/or HER2, the triple-negative tumors all cluster together. When the correlation coefficient to each of the molecular subtypes was calculated for the basal-like tumors as defined by Hu *et al*. [3], 91% of the triple-negative tumors showed the highest correlation coefficient to the basal-like centroid. All 4 tumors that did not show the highest correlation coefficient to the basal-like centroid, did show a correlation coefficient >0.1. However, a higher correlation coefficient was found with the normal epithelial subtype for each of those cases. From these results we conclude that the triple-negative tumors can be considered to be the same set of tumors that are defined as basal-like subtype tumors based on gene-expression profiling.

An important aim of our analyses was to explore how homogeneous the overall gene-expression profile is within the group of basal-like tumors; and whether it is possible to identify subsets of tumors defined by distinct differences in gene-expression patterns, including subsets associated with a low risk of developing distant metastases. For this purpose we also characterized the tumors in our study for the expression of ER, PR, HER2, EGFR, KIT and KRT5/6 [7,8], and for histopathological features including the presence of central fibrosis [26-29] and lymphocytic infiltrate [26,30-33].

We show that 5 subgroups of basal-like tumors can be identified based on the overall gene-expression profile. This indicates that basal-like tumors are not a homogeneous sub-group of breast carcinomas. We have denominated several known gene clusters, but these only partly explained the subgroup formation. The more prominent gene clusters that drive clustering into the main partitioning of the tumors could not be identified as a uniform biological mechanism.

Unfortunately, we were unable to identify a strong prognostic gene-expression profile in our study. However, there was a trend towards an improved metastasis-free survival for tumors with increased expression of interferon-regulated and immunoglobulin genes. This increased expression of immunoglobulin and interferon-related genes is likely to be the result of the presence of a lymphocytic infiltrate in the tumor. Galon *et al*. have recently described similar effects of the prognostic value of the adaptive immune response in controlling the growth and recurrence of colorectal cancer [34]. They characterized the tumor-infiltrating immune cells in a large cohort of human colorectal cancers by gene-expression profiling and immunohistochemical staining. They found that tumors with increased lymphocytic infiltrate and high expression levels of genes involved in immune response were associated with favorable prognosis.

In breast cancer the infiltration of stromal lymphocytes into the tumor is reported to be predominantly present in ER-negative breast carcinomas [30-33] and even more specific for the basal subtype [26,30]. We showed that lymphocytic infiltrate has the potential for further discriminating between tumors with good and poor prognosis within this group of triple-negative tumors. In addition we showed that central fibrosis is also a prognostic factor, and in order to verify that this effect is not just reflecting proliferation [26-29] we performed a multivariate analysis, showing that central fibrosis is a risk factor independent of the mitotic count (data not shown).

Another subdivision of basal-like tumors into prognostic groups has been suggested by Agoff *et al*., who showed that expression of AR in ER-negative tumors is associated with relatively good survival (log rank = 0.049) [35]. Previously, Farmer *et al*. described an apocrine tumor subtype based on gene-expression profiling that is characterized by AR expression, which distinguished these tumors from other basal-like tumors, but with similar poor survival [24]. In our study, overexpression of the apocrine/AR-related gene cluster is clearly visible in the tumor dendogram, but for the small number of AR-positive tumors, there is no association with outcome (metastasis-free or overall survival) as compared with the AR-negative tumors (data not shown).

An important clinical rationale for studying triple-negative tumors is to try to identify novel therapeutic targets within this subgroup, as these tumors do not respond to ER- and HER2-targeted therapies. EGFR-targeted therapy is an option for some of these tumors [8,26,33,36], but our data shows that 73% of the basal-like tumors are EGFR negative. It has been found that *BRCA1*-mutated breast carcinomas are almost always triple negative [37]; it would be interesting to know whether *BRCA1 *mutated tumors have a distinct gene-expression profile that distinguished them from the other basal type tumors. We do not have data on the BRCA1 status of the 97 tumors in our study, making it impossible to address this issue.

Specific subgroups of triple-negative tumors may be formed by medullary and atypical medullary cancers [12,37-39]. Medullary carcinoma was first described by Ridolfi *et al*. [40], and is characterized by distinct histological features, including a dense lymphocytic infiltrate, pushing margins, strong nuclear pleomorphism and a syncytial growth pattern. It has been shown that there is poor inter-observer agreement between pathologists, and that tumors that exhibit all the required histological features to make a diagnosis of medullary carcinoma is extremely rare [41-43]. None of the tumors in our study was classified as medullary carcinoma.

Bertucci *et al*. have tried to overcome this problem by differentiating medullary cancers from other ductal cancers by gene-expression profiling [30]. Using supervised classification they identified a gene list of 534 genes that could accurately classify 19 out of 21 medullary carcinomas. Unfortunately their sample size was limited, and they did not perform a validation of their signature on an independent dataset; therefore it is questionable whether they have found a robust medullary carcinoma signature or merely a classifier that defined other 'medullary like' features, such as dense lymphocytic infiltrate.

Livasy *et al*. have recently compared 23 basal-like tumors to 33 non-basal-like tumors (defined by gene-expression profiling), and found that basal-like tumors are significantly associated with high mitotic counts; they found geographic necrosis in 74% of the basal-like tumors, pushing margins in 61% and lymphocytic infiltrate in 56% [26]. Furthermore these basal-like tumors were immunophenotypically negative for ER and HER2 in 100%, and positive for KRT5/6 in 61%. These results are concordant with our findings.

Van de Rijn *et al*. have studied more than 600 breast cancer tumors with immunohistochemical analysis of the expression of basal keratins 5/6 and 17 [44]. They found that 16% of the cases stained positive for KRT5/6 and/or 17, and that these tumors had a worse prognosis compared with tumors that stained negative for either KRT5/6 or 17. In a subsequent study, Van de Rijn and coworkers showed in a series of 930 tumors that 16% stained positive for keratin 5/6 and/or 17 and that these basal keratin-positive tumors were associated with poor outcome in lymph-node-positive breast cancer patients, but not in tumors without lymph node metastases [8].

We have also tested a previously published gene signature that was constructed to predict metastasis-free survival in ER-negative tumors [14]. Wang *et al*. showed with a ROC curve that 16 genes were sufficient to have 100% performance in predicting metastasis-free survival in ER-negative patients. We used hierarchical cluster analysis to divide the tumors in our study into two groups based on the expression of these 16 genes, but were unable to show a correlation with outcome. Of note, 14% of the 35 ER-negative tumors in the study by Wang *et al*. were PR positive; the HER2 status of these 35 tumors was not provided. Therefore, the 35 tumors from the study by Wang *et al*. are not all triple negative.

## Conclusion

Basal-like tumors can be reliably defined by immunohistochemistry (ER-, PR- and HER2-negative; triple negative). These triple-negative tumors can be subdivided in at least five distinct subgroups of breast carcinomas that differ in their overall gene-expression profile. Although gene-expression profiling does not define prognostic subgroups, classical histological factors do appear to be associated with prognosis: the development of distant metastases was associated with the presence of central fibrosis and a small amount of lymphocytic infiltrate.

## Abbreviations

AR = androgen receptor; CI = confidence interval; CMF = cyclophosphamide, methotrexate and fluorouracil; DCIS = ductal carcinoma *in situ*; EGFR = epidermal growth factor receptor; ER = estrogen receptor; FU = follow up; GO = gene ontology; HR = hazard rate; IDC (nos) = infiltrating ductal carcinoma (not otherwise specified); ILC = infiltrating lobular carcinoma; KRT = keratin; LCIS = lobular carcinoma *in situ*; PR = progesterone receptor.

## Competing interests

These authors declare that they have no competing interests.

## Authors' contributions

BK had full access to all of the data in the study and takes responsibility for the integrity of the data and the accuracy of the data analysis, was responsible for the study concept and design, acquired the data, analysed and interpreted the data, drafted the manuscript, critically revised the manuscript for important intellectual content, and was responsible for the statistical analysis. MK acquired the data, analysed and interpreted the data, drafted the manuscript, critically revised the manuscript for important intellectual content, and was responsible for the statistical analysis. HH helped in the acquirement of the data and critically revised the manuscript. BW helped in the acquirement of the data and critically revised the manuscript for important intellectual content. HP acquired the data, analysed and interpreted the data. HB critically revised the manuscript for important intellectual content. MV had full access to all of the data in the study and takes responsibility for the integrity of the data and the accuracy of the data analysis, was responsible for the study concept and design, acquired the data, analysed and interpreted the data, drafted the manuscript, critically revised the manuscript for important intellectual content, and obtained funding for the study. All authors read and approved the final manuscript.

## Supplementary Material

Additional file 1 Figure showing hierarchical cluster analysis with 97 triple-negative tumors combined with 102 invasive breast carcinomas, which were not selected on the basis of their triple-negative status, using the 293 intrinsic-gene list. Click here for file

Additional file 2 A figure showing (a) hierarchical cluster analysis of 97 triple-negative tumors and 7,770 genes. Gene clusters as described in the text are indicated with colors on the right representing the position of the cluster, which are in part presented in parts b-h. (b) Immunoglobulin cluster. (c) Interferon-regulated cluster. (d) Keratin cluster. (e) Proliferation cluster. (f) Ion transporter activity cluster. (g) Apocrine-basal cluster. (h) Apocrine-luminal cluster. Click here for file

Additional file 3 A figure showing the Kaplan-Meier curve of distant-metastasis-free survival in 71 patients with triple-negative breast tumors. Click here for file

Additional file 4 Table showing the results of the supervised analysis using SAM software of 71 triple-negative tumors. Click here for file

Additional file 5 A figure showing validation of the Rotterdam ER-negative prognosis signature. (a) Hierarchical cluster analysis of 71 triple-negative tumors with the 16-prognosis-gene signature as described by Wang *et al*. [14]. The black and white bar between the tumor dendogram and the gene-expression matrix represents tumors that develop distant metastases (black box) and those that remain metastasis free (white box). (b) Metastasis-free survival of 71 patients with triple-negative breast carcinomas, comparing the left branch to the right branch of the hierarchical cluster analysis as depicted in part a. Click here for file
