# Supplementary material for: Gene expression profiling and histopathological characterization of triple-negative/basal-like breast carcinomas
Source: Breast Cancer Res. 2007 Oct 2;9(5):R65. doi: 10.1186/bcr1771 (PMC2242660; doi:10.1186/bcr1771)

Samples color-coded in red indicate the triple negative tumors (n=97).

Samples color-coded in green and blue indicate the invasive breast carcinomas, that were not selected based on their triple negative status (n=102). The green samples represent the 19 additional triple-nagative samples that cluster with the 97 selected triple-negative tumors.

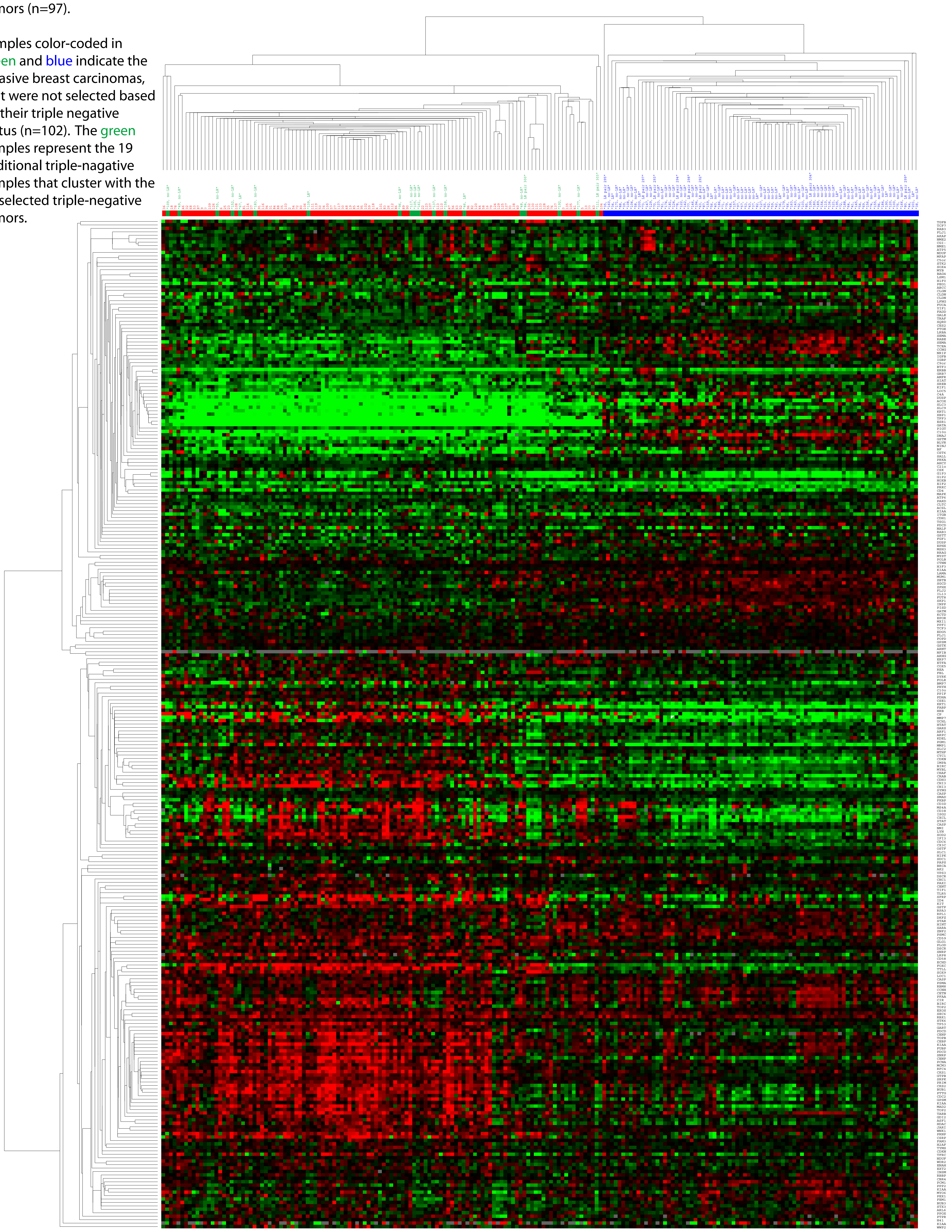

Supplement: Additional file 1 — Figure showing hierarchical cluster analysis with 97 triple-negative tumors combined with 102 invasive breast carcinomas, which were not selected on the basis of their triple-negative status, using the 293 intrinsic-gene list. [file bcr1771-S1.pdf]
